# Gesundheit und Studienpensum von Studierenden: Ergebnisse eines Gesundheitssurveys an der Universität Kassel

**DOI:** 10.1007/s11553-023-01035-6

**Published:** 2023-04-28

**Authors:** Alfons Hollederer

**Affiliations:** Fachbereich 01 Humanwissenschaften, Institut für Sozialwesen, Professur Theorie und Empirie des Gesundheitswesens, Universität Kassel, Arnold-Bode-Str. 10, 34127 Kassel, Deutschland

**Keywords:** Gesundheitszustand, Psychische Gesundheit, Menschen mit Behinderungen, Studentisches Gesundheitsmanagement, Basiserhebung, Health status, Mental health, Disabled Persons, Student Health Services, Baseline survey

## Abstract

**Einleitung:**

Über Gesundheit und Krankheit von Studierenden ist relativ wenig bekannt und die Auswirkungen auf den Studienerfolg sind kaum erforscht. Das Ziel der Studie ist, Assoziationen zwischen dem Gesundheitszustand von Studierenden und dem absolvierten Studienpensum zu analysieren.

**Methoden:**

Es nahmen 3330 von 23.699 immatrikulierten Studierenden der Universität Kassel an der freiwilligen Befragung („computer assisted web interviews“) im März 2022 teil. Die Rücklaufquote betrug 14,1 % unter Bedingungen der Coronapandemie.

**Ergebnisse:**

Ihren allgemeinen Gesundheitszustand bewerteten 80,5 % der Studierenden als sehr gut oder gut. Es gab signifikante Unterschiede zwischen Männern und Frauen beim allgemeinen Gesundheitszustand (84,4 % vs. 78,6 %) sowie beim „global activity limitation indicator“ (GALI). Frauen waren häufiger als Männer seit mindestens 6 Monaten bei alltäglichen Aktivitäten gesundheitsbedingt stark eingeschränkt (3,2 % vs. 2,6 %) oder mäßig eingeschränkt (9,6 % vs. 5,7 %). Auffällig waren die berichteten Prävalenzraten von psychischen Erkrankungen in den letzten 12 Monaten. Sie lagen bei Frauen wesentlich höher als bei Männern (25,3 % vs. 15,4 %). 15,1 % der Studierenden gaben an, dass ihr absolviertes Studienpensum im Umfang „viel weniger“ den Vorgaben der Studienordnung ihres derzeitigen Studiengangs entsprach. Eine logistische Regressionsanalyse eruierte in multivariater Betrachtung, dass Studierende, die gesundheitsbedingt mäßig eingeschränkt waren, ein signifikant gesteigertes Odds Ratio von 1,56 (95 %-Konfidenzintervall [KI] 1,07–2,27) und Studierende mit starken Einschränkungen von 2,81 (95 %-KI 1,64–4,80) für ein viel zu geringes Studienpensum aufwiesen.

**Schlussfolgerung:**

Die Studie ermittelte enge Assoziationen zwischen Gesundheit und Studienpensum. Sie zeigt die Notwendigkeit von mehr Gesundheitsförderung und für ein Gesundheitsmanagement bei Studierenden mit Behinderungen und Gesundheitseinschränkungen auf.

## Abstract (en)

**Introduction:**

Relatively little is known about students’ health and illness. Their effects on academic success have hardly been researched. The aim of the study is to examine associations between health status and study load completed among students.

**Methods:**

Of 23,699 students enrolled at the University of Kassel, 3330 participated in the voluntary survey (computer-assisted web interviews) in March 2022. The response rate was 14.1% under conditions of the coronavirus disease 2019 (COVID-19) pandemic.

**Results:**

In all, 80.5% of students rated their general health status as very good or good. There were significant differences between men and women in terms of general health status (84.4% vs. 78.6%) and the global activity limitation indicator (GALI). Women were more often than men severely limited in usual activities due to health problems (3.2% vs. 2.6%) or limited but not severely (9.6% vs. 5.7%) for at least the past 6 months. Reported prevalence rates of mental illness were striking. They were higher in women than in men (25.3% vs. 15.4%). Furthermore, 15.1% of students reported that their study load completed was “much less” than required by the study regulations of current degree programs. A multivariate logistic regression analysis revealed that students who were limited in activities of daily life because of a health problem had a significantly increased odds ratio of 1.56 (95%-confidence interval [CI] 1.07–2.27) for a much lower study load, and those students who were severely limited had a significantly increased odds ratio of 2.81 (95%-CI 1.64–4.80).

**Conclusion:**

The study identified strong associations between health and study load. It points out the need not only for more health promotion but also for health management for students with disabilities and limitations due to health problems.

## Hintergrund

Die Hochschulbildung hat in Deutschland während der letzten Dekaden stark an Bedeutung gewonnen. Die Zahl der immatrikulierten Studierenden erreichte mit über 2,9 Mio. im Wintersemester 2021/22 ein Allzeithoch [32]. Eine Hochschule ist nicht nur ein Ort der Wissensvermittlung. Sie ist eine Lebenswelt, die nach dem biopsychosozialen Modell [39] die Gesundheit und Krankheit von Menschen, die sich darin aufhalten, beeinflussen kann. Die Ottawa-Charta zur Gesundheitsförderung der Weltgesundheitsorganisation (WHO) propagierte bereits 1986 die Schaffung von „gesundheitsförderlichen Lebenswelten“ [40], u. a. auch an Lernorten. Wenig später wurden Hochschulen als eigenes Setting der Gesundheitsförderung von der WHO anerkannt [37] und internationale Netzwerke gegründet [36]. Die Gesundheitsförderung bei Studierenden wurde in Deutschland mit dem Präventionsgesetz 2015 gestärkt und nach dem Lebensweltansatz im § 20a Abs. 1 SGB V als Aufgabe der gesetzlichen Krankenversicherung sowie in den Bundesrahmenempfehlungen der Nationalen Präventionskonferenz [24] adressiert. Umso verwunderlicher ist es, dass über Gesundheit und Morbidität bei Studierenden relativ wenig bekannt ist. Es fehlt eine systematische Gesundheitsberichterstattung [9].

Studierende gelten gemeinhin als gesunde Bevölkerungsgruppe, da jüngeres Alter und ein hohes Bildungsniveau epidemiologisch mit besserer Gesundheit assoziiert sind [11]. Erst in der letzten Zeit verdichteten sich Hinweise aus empirischen Studien, dass auch bei Studierenden zunehmend Gesundheitsprobleme auftreten. Eine wichtige Datenquelle ist die repräsentative Online-Erhebung „Gesundheit Studierender in Deutschland 2017“ von Grützmacher et al. [8]. 82 % der 6198 befragten Studierenden bewerteten ihren allgemeinen Gesundheitszustand als gut oder sehr gut. Das ist einerseits die weit überwiegende Mehrheit der Studierenden, andererseits ist dabei als wichtiger empirischer Befund zu beachten, dass dieser Prozentsatz unter den Vergleichswerten von mehreren nationalen Gesundheitssurveys des Robert Koch-Instituts (RKI) für diese Altersgruppe liegt. In der Repräsentativerhebung GEDA 2019/2020-EHIS [11] beschrieben 87 % der Frauen und 88 % der Männer in der Altersgruppe von 18 bis 29 Jahre ihre allgemeine Gesundheit als sehr gut oder gut. Diese Gegenüberstellung lässt die Schlussfolgerung zu, dass Studierende entgegen den bisherigen Erwartungen keinen besseren Gesundheitszustand als andere Gleichaltrige aufweisen.

Im Rahmen der 21. Sozialerhebung des Deutschen Studentenwerks befragten Middendorff et al. [23] im Jahr 2016 insgesamt 55.219 Studierende in Deutschland. Davon gaben 12 % der Frauen und 10 % der Männer an, dass sie an mindestens einer gesundheitlichen Beeinträchtigung leiden, welche sich erschwerend auf das Studium auswirkt. Der Anteil beeinträchtigter Studierender ist gegenüber der Sozialerhebung 2012 von insgesamt 7 % auf 11 % gestiegen. Wie eine Sonderauswertung des Studierendenwerks Kassel bei 395 teilgenommenen Studierenden zeigte, war der entsprechende Anteil für die Universität Kassel mit 15 % überproportional hoch [35].

Vergleichsanalysen mit Krankenkassendaten der Techniker Krankenkasse kamen dagegen zu einem differenzierten Bild für das Versorgungsgeschehen 2013/2014 [7]. Die Inanspruchnahme der ambulanten ärztlichen Versorgung sowie die Arzneimittelverordnungen waren bei Studierenden geringer als bei jungen Erwerbstätigen. Auffällig war jedoch, dass Studierende häufiger als junge Erwerbstätige Kontakte zu psychologischen Psychotherapeuten und Psychotherapeutinnen aufnahmen (4,3 % vs. 2,4 %) und ihnen mehr Antidepressiva verordnet wurden. Die weiblichen Studierenden waren besonders stark betroffen.

Studierende sind außerdem keine sozial homogene Gruppe [17]. Die Gesundheit und das Gesundheitsverhalten unterscheiden sich innerhalb der Studierendenschaft erheblich nach dem subjektiven Sozialstatus [3].

Den Zusammenhang von Gesundheitsbeeinträchtigung und Studium untersuchten Poskowsky et al. [26] in der Studie „Beeinträchtigt Studieren – BeSt II“ in 2016/17. 62 % der knapp 21.000 teilnehmenden Studierenden mit studienerschwerenden gesundheitlichen Beeinträchtigungen schätzten die Auswirkungen ihrer Beeinträchtigungen auf das Studium als stark oder sehr stark ein.

Dieser Befund wird durch eine Analyse zu den Absolventenstudien von Plasa [25] bestätigt. Sie zeigte, dass gesundheitliche Beeinträchtigungen einen wichtigen Prädiktor für eine verlängerte Studiendauer und eine verzögerte Berufseinmündung darstellen.

Andere Forschungsarbeiten weisen ebenfalls auf wechselseitige Zusammenhänge zwischen der individuellen Gesundheit von Studierenden und dem akademischen Erfolg hin [4, 34].

In der Coronapandemie veränderten sich die Studienbedingungen an deutschen Hochschulen aufgrund von Länderverordnungen mit Kontaktbeschränkungen, Hygienevorschriften und digitaler Online-Distanzlehre massiv [13]. Zeitgleich sind offenbar die Sorgen und Ängste von Studierenden angestiegen. In einer Erhebung an vier deutschen Universitäten [31] befürchtete rund ein Drittel der Studierenden, selbst mit COVID-19 („coronavirus disease 2019“) infiziert zu werden und gut drei Viertel waren besorgt, dass Angehörige nach der Ansteckung schwer erkranken könnten. Die coronabedingten Sorgen waren in dieser und einer anderen Studie [6] mit depressiven Symptomen assoziiert. Eine weitere Befragung von 28.600 Studierenden [41] ergab im Sommersemester 2020 stärkere Belastungen für vulnerable Studierendengruppen. Studierende mit gesundheitlichen Beeinträchtigungen, COVID-19-Risikogruppen und Studierende mit Kind berichteten wesentlich häufiger als die übrigen Studierenden, dass sie Ängste vor COVID-19-Infektionen hatten und sich mehr gestresst fühlten.

In der Wissenschafts- und Hochschulforschung werden vielfältige Gründe für Studienerfolg und Studienabbruch diskutiert. Hingegen werden die Zusammenhänge mit Gesundheitseinschränkungen von Studierenden sowie möglichen Unterstützungsangeboten nach einer Überblicksarbeit von Römhild und Hollederer [29] wenig analysiert. Es besteht ein Forschungsdefizit in Verbindung mit Studienerfolg. Die vorliegende Analyse an der Universität Kassel zielt darauf ab, Assoziationen von Gesundheit und Studienpensum zu identifizieren. Die Universität Kassel deckt mit ihren Fachbereichen ein weites Spektrum mit über 150 Studiengängen ab.

## Methoden

Im März 2022 wurden 23.699 Studierende der Universität Kassel, die im Wintersemester 2021/22 eingeschrieben waren, zu einer Online-Befragung („computer assisted web interview“) eingeladen. Die Studierenden erhielten eine E‑Mail mit Motivationsanschreiben und verschlüsseltem Zugangslink auf die E‑Mail-Accounts, welche bei Immatrikulation von der Universität eingerichtet wurden. Die Teilnahme war freiwillig. Während des Befragungszeitraums erfolgten mehrere Erinnerungen. Als Motivationsanreiz wurden Artikel aus dem Universitätsshop verlost.

Der Fragebogen bestand aus Modulen zu soziodemografischen Angaben, zur Studiensituation, Behinderung und Gesundheitsvariablen. Im Vorfeld der Erhebung wurde ein Pretest bei 10 Studierenden durchgeführt. Die selbst wahrgenommene Gesundheit wurde über die Frage „Wie ist Ihr Gesundheitszustand im Allgemeinen?“ und Antwortoptionen auf einer 5er-Skala im Likert-Format operationalisiert („sehr gut; gut; mittelmäßig; schlecht; sehr schlecht“). Das Item gilt als geeigneter Proxy-Indikator für den objektiven Gesundheitszustand und wird von der Weltgesundheitsorganisation für den Einsatz in Gesundheitssurveys empfohlen [38].

Ein weiteres Item erfasste das Vorliegen einer chronischen Erkrankung über die Frage „Haben Sie eine oder mehrere lang andauernde, chronische Krankheiten?“ („ja; nein; ich weiß nicht“) aus der GEDA-Studie [11]. Es wurde der Ausfüllhinweis gegeben, dass damit Krankheiten oder gesundheitliche Probleme gemeint sind, die mindestens 6 Monate andauern oder voraussichtlich andauern werden.

Zur Erfassung der Krankheiten in den letzten 12 Monaten wurde eine Auswahlliste vorgelegt, die sich an relevanten Kapiteln und Einzeldiagnosen der Internationalen statistischen Klassifikation der Krankheiten und verwandter Gesundheitsprobleme (ICD-10 GM) orientiert („ja/nein“). Zusätzlich wurde das Vorhandensein von Teilleistungsstörungen und einer Bewegungsbeeinträchtigung abgefragt.

Der „global activity limitation indicator“ (GALI) eruiert mit 3 Fragen die Gesundheitseinschränkungen bei alltäglichen Aktivitäten, die schon mindestens 6 Monate anhalten. Der Indikator wird von der Europäischen Union für das Monitoring für die Europäische Strategie zugunsten von Menschen mit Behinderungen eingesetzt und konzeptionell als guter Proxy-Indikator für die Messung von Behinderung angesehen [5]. Eingangs wurde die Frage „Sind Sie dauerhaft durch ein gesundheitliches Problem bei Tätigkeiten des normalen Alltagslebens eingeschränkt?“ gestellt. Wenn die Frage bejaht wurde, schloss sich die Nachfrage an: „Wie stark sind Sie bei Tätigkeiten des normalen Alltagslebens eingeschränkt?“ („wenig eingeschränkt“; „stark eingeschränkt“). Die letzte Frage klärte, ob die Einschränkung schon länger als 6 Monate andauert. Aus diesen Antworten lassen sich die drei Kategorien des GALI „stark eingeschränkt“, „mäßig eingeschränkt“ und „nicht eingeschränkt“ bilden.

Außerdem kam ein Messinstrument für die Wahrnehmung von Studienanforderungen (MWS) von Jänsch und Bosse [19] in einer gekürzten Fassung von neun Fragen mit einer 5‑stufigen Zustimmungsskala zum Einsatz. Es erfasste, wie schwer der Umgang mit verschiedenen Studienanforderungen im Wintersemester 2021/2022 fiel. Eine Frage betraf das soziale Klima. Drei weitere Fragen erhoben die Bewältigung der Studienorganisation (Lehrangebot, Finden passender Informations- und Beratungsangebote, Umgang mit Rahmenbedingungen). Zwei Fragen erkundeten den Umgang mit Leistungsdruck und Prüfungsbedingungen. Die letzten drei Fragen eruierten Lernaktivitäten (Strukturierung von Lernaktivitäten, Bewältigung des Lernstoffs und Einschätzung der Belastbarkeit).

Das bisher absolvierte Studienpensum wurde mit einem übernommenen Einzelitem des nationalen Bildungspanels [21] ermittelt („Inwieweit entspricht Ihr bisher absolviertes Studienpensum [z. B. Anzahl besuchter Lehrveranstaltungen/Kurse, Anzahl erfolgreich absolvierter Studien‑/Prüfungsleistungen, erhaltene Leistungspunkte etc.] den Vorgaben der Studienordnung Ihres derzeitigen Studiengangs?“). Die fünf Antwortoptionen wurden für die Regressionsanalyse dichotomisiert nach „viel weniger“ vs. „etwas weniger“/„etwa genau so viel“/„etwas mehr“/„viel mehr“.

Der Untersuchungsansatz verwendet deskriptive Statistik und Korrelationsanalysen. In einem logistischen Regressionsmodell wurde die Wahrscheinlichkeit eines viel zu geringen Studienpensums in multivariater Betrachtung analysiert. Die binäre logistische Regression berechnet die Wahrscheinlichkeit des Eintreffens eines Ereignisses in Abhängigkeit von den Werten der Prädiktoren und weist 95 %-Konfidenzintervalle aus. Zur Berechnung der Signifikanzen wurde die Wald-Statistik verwendet (*p*-Werte < 0,05). Die Zugehörigkeit zu der mit „1“ kodierten Gruppe wird geschätzt, wobei die Ausprägung der abhängigen Variable „0“ und „1“ beträgt. Die Odds Ratio (OR, „Chancenverhältnis“) ist ein Maß dafür, um wieviel größer die Wahrscheinlichkeit eines Ereignisses in der Gruppe mit bestimmten Merkmalen im Vergleich zur Gruppe ohne diese Merkmale ist. Sie sagt aus, wie stark dieser Zusammenhang ist.

Die Daten der Online-Befragung wurden mittels programmiertem Online-Survey (LimeSurvey GmbH, Hamburg) erhoben und in das Statistikprogramm IBM SPSS zur quantitativen Analyse exportiert. Die Berechnungen wurden mit IBM SPSS-Statistics Version 28 (USA, New York) durchgeführt.

## Ergebnisse

### Rücklaufquoten und Soziodemografie

Insgesamt nahmen 3330 Studierende von 23.699 eingeschriebenen Studierenden der Universität Kassel an der freiwilligen Befragung teil. Die Rücklaufquote betrug 14,1 % und entsprach einer üblichen Response von Studierenden in der Coronapandemie [28]. 71 nichtteilnehmende Studierende beantworteten eine kurze Non-response-Befragung. Darunter gaben zwei Drittel Zeitgründe als Grund für die Nicht-Teilnahme an.

In der Mitte des Fragebogens (nach den Gesundheitsangaben) gab es auffällig viele Abbrüche der Beantwortung bei einem Fragenblock nach den exakten Leistungspunkten sowie Studien- und Prüfungsleistungen.

Die Tab. 1 weist die Struktur der Studierenden an der Universität Kassel nach soziodemografischen und studienbezogenen Merkmalen aus. Da die Universität Kassel eine offizielle Studierendenstatistik führt, können für einzelne Merkmale die Rücklaufquoten berechnet werden. Die Tab. 1 zeigt auf, dass es eine überproportional hohe Response bei Studierenden im 1. Fachsemester und bei weiblichen Studierenden gab. Letzteres könnte darauf zurückzuführen sein, dass Frauen sich i. Allg. mehr für Gesundheitsthemen als Männer interessieren. Dagegen lagen die Rücklaufquoten insbesondere bei Studierenden der lehrerbildenden Studiengänge und bei ausländischen Studierenden etwas unter dem Durchschnitt. Die Rücklaufquote von den Studierenden aus dem Fachbereich 01 „Humanwissenschaften“ war besonders hoch, die der Kunsthochschule verhältnismäßig niedrig.

**Tab. 1:** Rücklaufquoten nach Studienmerkmalen der Studierenden der Universität Kassel im Wintersemester 2021/2022

Merkmale	Interviewte Studierende		Alle Studierende		Rücklaufquote
	Anzahl (*n*)	%	Anzahl (*n*)^a^	%	%
*Insgesamt*	3.330	100,0	23.699	100,0	14,1
Darunter:					
Frauen	1.950	58,6	11.860	50,0	16,4
Ausländische Staatsangehörigkeit	352	10,6	3.172	13,4	11,1
1. Fachsemester	812	24,4	4.443	18,7	18,3
*Angestrebter Abschluss:*					
Lehrerbildende Studiengänge	505	15,2	5.058	21,3	10,0
Bachelor	1.694	50,9	11.280	47,6	15,0
Master	983	29,5	6.456	27,2	15,2
Andere (Künstlerischer Abschluss, Diplom, Sonstige)	145	4,4	905	3,8	16,0
*Fachbereiche (FB):*					
FB 01 Humanwissenschaften	536	16,1	2.664	11,3	20,1
FB 02 Geistes- und Kulturwissenschaften	373	11,2	2.204	9,3	16,9
FB 05 Gesellschaftswissenschaften	334	10,0	2.303	9,7	14,5
FB 06 Architektur-Stadtplanung-Landschaftsplanung	270	8,1	1.500	6,3	18,0
FB 07 Wirtschaftswissenschaften	705	21,2	5.994	25,3	11,8
FB 10 Mathematik und Naturwissenschaften	261	7,8	2.659	11,2	9,8
FB 11 Ökologische Agrarwissenschaften	173	5,2	1.127	4,8	15,4
FB 14 Bauingenieur- und Umweltingenieurwesen	187	5,6	1.255	5,3	14,9
FB 15 Maschinenbau	180	5,4	1.424	6,0	12,6
FB 16 Elektrotechnik/Informatik	224	6,7	1.603	6,8	14,0
Kunsthochschule	87	2,6	928	3,9	9,4

^a^Datenquelle: Akademisches Managementinformationssystem der Universität Kassel (AKADEMIS) vom 14.07.2022

Das Durchschnittsalter der erfolgreich befragten Studierenden betrug 25,9 (SE = 0,11) Jahre. Die Frauen waren im Mittel etwas jünger als die Männer (25,6 vs. 26,2 Jahre). Rund zwei Drittel der Studierenden gingen neben dem Studium im letzten Semester einer Erwerbstätigkeit nach, wie in Tab. 2 zu sehen ist.

**Tab. 2:** Gesundheits- und studienbezogene Variablen nach Geschlecht

		Männer	Frauen	*p*-Wert^a^
*I. Gesundheitsvariablen*^b^				
Allgemeiner subjektiver Gesundheitszustand	*n*	1.249	1.916	–
	1 sehr gut	36,8 %	28,9 %	< 0,001
	2 gut	47,6 %	49,7 %	
	3 mittelmäßig	13,1 %	17,5 %	
	4 schlecht	2,2 %	3,5 %	
	5 sehr schlecht	0,2 %	0,3 %	
Chronische Krankheit (lang andauerndes gesundheitliches Problem)	*n*	1.244	1.908	–
	Ja	21,5 %	29,8 %	< 0,001
	Nein	78,5 %	70,2 %	
Amtlich anerkannte Behinderung	*n*	1.240	1.900	*–*
	Ja	3,4 %	2,2 %	< 0,05
	Nein	96,6 %	97,8 %	
Teilleistungsstörung (Legasthenie, Dyskalkulie u. a.)	*n*	1.235	1.901	*–*
	Ja	5,3 %	4,5 %	0,310
	Nein	94,7 %	95,5 %	
Bewegungsbeeinträchtigung (u. a. beim Gehen, Stehen, Greifen, Tragen)	*n*	1.238	1.899	*–*
	Ja	4,2 %	4,1 %	0,841
	Nein	95,8 %	95,9 %	
Krankheiten in den letzten 12 Monaten (Mehrfachnennungen)	*n*	1.222	1.876	–
	Keine Krankheiten	60,7 %	50,4 %	< 0,001
	(a) Krankheiten des Auges, darunter:	5,5 %	6,7 %	0,184
	Stark sehbehindert oder blind	0,6 %	0,6 %	0,814
	(b) Krankheiten des Ohres, darunter:	4,3 %	5,3 %	0,201
	Hochgradig schwerhörig oder gehörlos	0,3 %	0,2 %	0,543
	(c) Psychische Erkrankungen, darunter:	15,4 %	25,3 %	< 0,001
	Diagnostizierte Angststörung	6,7 %	11,5 %	< 0,001
	Diagnostizierte Depression	10,6 %	17,0 %	< 0,001
	Diagnostizierte Essstörung	1,4 %	4,8 %	< 0,001
	(d) Krankheiten des Nervensystems	2,9 %	4,5 %	< 0,05
	(e) Krankheiten des Verdauungssystems	9,7 %	16,0 %	< 0,001
	(f) Krankheiten des Muskel-Skelett-Systems	5,7 %	8,4 %	< 0,01
	(g) Krankheiten des Atmungssystems	11,1 %	12,8 %	0,149
	(h) andere Krankheiten	12,9 %	17,9 %	< 0,001
„Global activity limitation indicator“ (GALI)	*n*	1.242	1.898	*–*
	Keine alltäglichen Einschränkungen	91,7 %	87,2 %	< 0,001
	Mäßig eingeschränkt	5,7 %	9,6 %	
	Stark eingeschränkt	2,6 %	3,2 %	
*II. Studienbezogene Variablen*				
Studienpensum entspricht Studienordnung	*n*	1.120	1.723	–
	1 viel weniger	17,7 %	13,3 %	< 0,01
	2 etwas weniger	30,3 %	28,0 %	
	3 etwa genau so viel	33,2 %	40,4 %	
	4 etwas mehr	13,2 %	15,0 %	
	5 viel mehr	5,6 %	3,3 %	
Schwierigkeiten mit Studienanforderungen	*n*	958	1.497	–
	Keine Schwierigkeiten im Studium	29,9 %	24,7 %	< 0,01
	Schwierigkeiten bei mindestens 1 Anforderung	70,1 %	75,3 %	
Finanzielle Belastung der Studierenden und Familie bis Studiumsende	*n*	958	1.497	–
	Gar nicht/kaum/etwas	70,5 %	64,0 %	< 0,001
	Ziemlich/sehr	29,5 %	36,0 %	
Erwerbstätig (neben Studium)	*n*	941	1.493	–
	Nein	32,1 %	32,0 %	0,968
	Ja	67,9 %	68,0 %	

^a^*p*-Wert basiert auf χ^2^-Test bzw. Mann-Whitney-U-Test

^b^Wegen zu kleiner Fallzahlen und Datenschutz wurde diverses Geschlecht in der Tabelle nicht ausgewiesen

### Krankheitsprävalenzraten und Gesundheitsunterschiede zwischen Männern und Frauen

Die Tab. 2 veranschaulicht die gesundheits- sowie die studienbezogenen Variablen. Sie enthält detaillierte Informationen zur Gesundheit und zu den Krankheitsprävalenzraten bei den befragten Studierenden der Universität Kassel. Die Stratifizierung nach Männern und Frauen legt dabei vielfältige signifikante Gesundheitsunterschiede offen. Entsprechend dem Durchschnittsalter wird der allgemeine Gesundheitszustand von den meisten Studierenden erwartungsgemäß als positiv bewertet. Insgesamt schätzten 80,5 % der Studierenden ihren allgemeinen Gesundheitszustand als sehr gut oder gut ein. Die Männer berichteten signifikant häufiger einen sehr guten oder guten allgemeinen Gesundheitszustand als die Frauen (84,4 % vs. 78,6 %). Am entgegengesetzten Pol der Antwortskala stuften dennoch 3,5 % der Studierenden ihren allgemeinen Gesundheitszustand als schlecht oder sehr schlecht ein.

Ein weiteres Gesundheitsitem fragte nach dem Vorhandensein von chronischen Krankheiten. Hier antwortete gut ein Viertel (27,0 %) der Studierenden, dass bei ihnen eine chronische Krankheit oder ein lang andauerndes gesundheitliches Problem vorlag. Der Anteil der Frauen mit chronischen Erkrankungen betrug 29,8 % und übertraf in statistisch signifikanter Weise den Anteil der Männer von 21,5 %.

Eine sehr kleine, aber in der Gesundheitsperspektive relevante Gruppe von 2,8 % gab in der Befragung eine amtlich anerkannte Behinderung an (Tab. 2). Sie wird von den Versorgungsämtern nach versorgungsmedizinischen Kriterien auf Antrag hin festgestellt. Bei etwas mehr als der Hälfte (54,1 %) wurde ein Grad der Behinderung (GdB) von mindestens 50 und damit eine Schwerbehinderung zuerkannt. Die Schwerbehindertenquote in der Studierendenschaft ist beachtenswert, liegt aber insgesamt unter dem Bundesdurchschnitt dieser Alterskohorten [33].

Ein Einzelitem erkundigte sich nach den Teilleistungsstörungen. Darunter fallen Störungen wie Legasthenie oder Dyskalkulie. Davon waren nach den Selbstauskünften insgesamt 4,9 % der Studierenden betroffen.

Auffällig ist, dass der Anteil der Männer mit sehr guter oder guter subjektiver Gesundheit überproportional hoch ist, sie aber im Vergleich zu den Frauen prozentual sowohl mehr Behinderungen (3,4 % vs. 2,2 %) als auch häufiger Teilleistungsstörungen (5,3 % vs. 4,5 %) aufwiesen.

Der Survey erhob außerdem die Krankheiten, die im letzten Jahr auftraten, nach verschiedenen Diagnosegruppen (Tab. 2). Nach den Selbstauskünften litten fast die Hälfte bzw. 46,0 % der Studierenden in den letzten 12 Monaten an Krankheiten (Männer: 39,3 %; Frauen 49,6 %). Die größte Hauptdiagnosegruppe bildeten bemerkenswerterweise psychische Erkrankungen, die 22,0 % der Studierenden nannten. Davon berichteten die Frauen wesentlich häufiger als die Männer, dass sie eine psychische Erkrankung in den letzten 12 Monaten hatten (25,3 % vs. 15,4 %). Weitere Zusatzfragen eruierten ausgewählte Diagnosen innerhalb der Gruppe der psychischen Erkrankungen (Tab. 2). Darunter dominierten diagnostizierte Depressionen und Angststörungen. Ein kleiner, aber bemerkenswerter Teil der Studierenden offenbarte diagnostizierte Essstörungen.

Wie der Forschungsstand nahelegt [31, 41], dürfte sich im Beobachtungszeitraum die Coronapandemie besonders auf die psychische Gesundheit der Studierenden negativ ausgewirkt haben. Die Krankheiten des Atmungssystems, die normalerweise das Arbeitsunfähigkeitsgeschehen stark beeinflussen, folgen erst mit großem Abstand in der Häufigkeit der Nennungen.

Drei weitere Gesundheitsfragen richteten sich auf das wichtige Seh- und Hörvermögen sowie die Mobilität der Studierenden. 0,6 % der Studierenden bejahten die Frage, ob sie stark sehbehindert oder blind seien. 0,2 % der Studierenden gaben an, hochgradig schwerhörig oder gehörlos zu sein. Weitere 4,2 % der Studierenden konstatierten eine Bewegungsbeeinträchtigung.

Entgegen den Erwartungen beurteilten ausländische Studierende ihren Gesundheitszustand signifikant besser als Studierende mit deutscher Staatsangehörigkeit. 85,3 % der ausländischen Studierenden bewerteten ihren allgemeinen Gesundheitszustand als sehr gut oder gut. Ausländische Studierende gaben in Relation zu deutschen Studierenden in signifikant geringerem Ausmaß sowohl chronische Krankheiten (14,2 %) als auch amtlich anerkannte Behinderungen (1,2 %) und Krankheiten in den letzten 12 Monaten (38,0 %) bzw. gesundheitlich bedingte Einschränkungen im Alltag (4,5 %) an. Möglicherweise sind diese Beobachtungen auf das Paradoxon eines „healthy migrant effect“ [27] zurückzuführen.

Entsprechend dem GALI gelten insgesamt 8,4 % der befragten Studierenden aufgrund ihres lang andauernden gesundheitlichen Problems bei alltäglichen Aktivitäten als mäßig eingeschränkt und weitere 3,1 % als stark eingeschränkt. Wie die Tab. 2 informiert, sind Frauen wesentlich stärker von diesen Gesundheitseinschränkungen betroffen als Männer.

### Zusammenhänge von Gesundheit und Studium

Von großem Interesse ist, inwieweit das bisher absolvierte Studienpensum (z. B. Anzahl besuchter Lehrveranstaltungen/Kurse, Anzahl erfolgreich absolvierter Studien‑/Prüfungsleistungen, erhaltene Leistungspunkte etc.) den offiziellen Vorgaben entsprach. 15,1 % der Studierenden gaben an, dass ihr derzeitiges Studienpensum viel geringer ausfällt als die Studienordnung ihres Studiengangs vorgibt. Wie die Tab. 2 darlegt, erreichten die Frauen auf der einen Seite wesentlich häufiger als die Männer das vorgegebene Studienpensum, zeigten auf der anderen Seite jedoch vermehrt Schwierigkeiten bei den Studienanforderungen an. Lediglich ein Viertel (24,7 %) der Frauen gab an, keine Schwierigkeiten bei den abgefragten typischen Anforderungen im Studium zu haben. Bei den Männern ist der analoge Anteil mit 29,9 % signifikant höher.

**Abb. 1:**
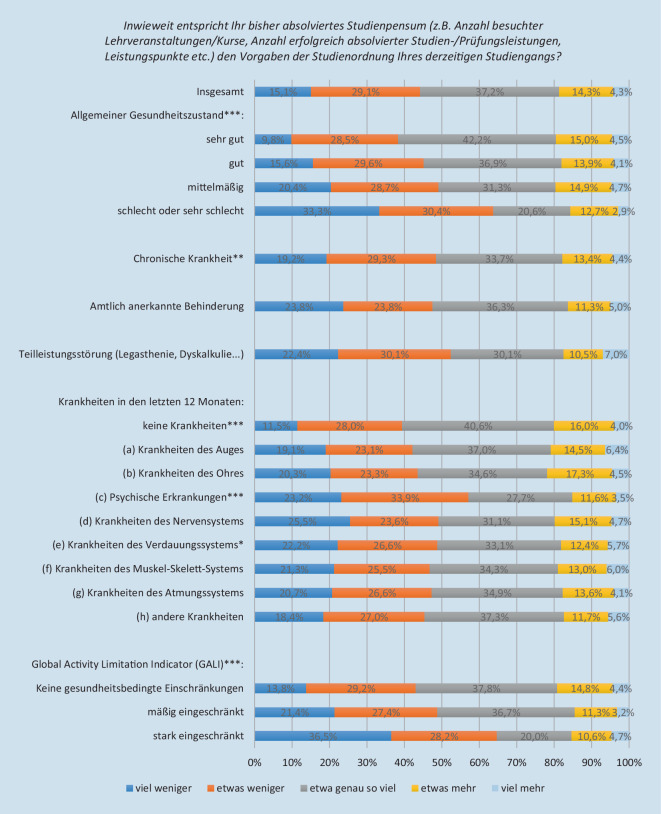
Gesundheit und Krankheit nach Studienpensum bei Studierenden. ***p < 0,001, **p < 0,01, *p < 0,05

Die Abb. 1 veranschaulicht die unterschiedlichen Antwortverteilungen zum absolvierten Studienpensum nach Gesundheitszuständen und Krankheiten. Sie demonstriert den Zusammenhang zwischen der Krankheitsschwere und dem Unterschreiten des vorgegebenen Studienpensums. Von den Studierenden, die gesundheitlich stark in den alltäglichen Aktivitäten eingeschränkt waren, sagte über ein Drittel (36,5 %) aus, dass ihr absolviertes Studienpensum viel weniger den Vorgaben entsprach. Je schlechter der subjektive Gesundheitszustand eingestuft wurde, desto kleiner fiel das Studienpensum im Mittel aus. Auffällig gering war das Studienpensum ebenfalls bei Studierenden, die in den letzten 12 Monaten unter psychischen Erkrankungen litten. Chronische Krankheiten korrelierten ebenfalls mit dem Studienpensum.

**Abb. 2:**
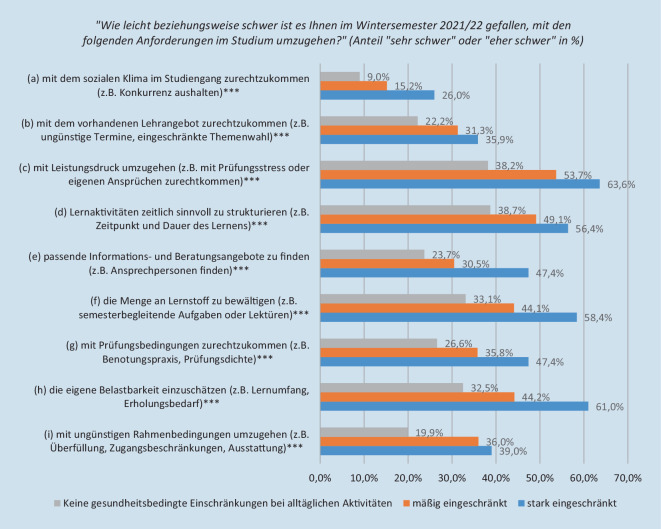
Anforderungen im Studium nach gesundheitsbedingten Einschränkungen bei alltäglichen Aktivitäten. ***p < 0,001; *p*-Werte basieren auf χ^2^-Test nach Pearson; *n* = 2582

Die Abb. 2 visualisiert die typischen Anforderungen im Studium und die Schwierigkeiten für die Studierenden im Umgang damit nach den drei Kategorien des GALI. Der Leistungsdruck stellt darunter die größte Herausforderung dar, welchen 40,2 % der Studierenden als eher schwer oder sehr schwer wahrnahmen. Studierende mit gesundheitlich bedingten Einschränkungen hatten wesentlich häufiger Schwierigkeiten im Umgang mit Leistungsdruck und Misserfolg, aber auch in der Studienorganisation und bei Lernaktivitäten. Je stärker die gesundheitlich bedingten Einschränkungen waren, umso häufiger gaben die Studierenden Schwierigkeiten in der Bewältigung der Studienanforderungen an.

Die Schwierigkeiten mit Studienanforderungen akkumulieren. 88,9 % der Studierenden mit gesundheitsbedingt starken Einschränkungen berichteten, dass ihnen bei mindestens einer der neun Anforderungen der Umgang eher schwer oder sehr schwer fiel, während der Prozentsatz bei Studierenden mit gesundheitsbedingt mäßigen Einschränkungen 83,0 % betrug. Die Anteile lagen signifikant über dem korrespondierenden Prozentsatz bei den Studierenden ohne gesundheitsbedingte Einschränkungen von 71,9 %.

### Gesundheit und Studienpensum

Die binäre logistische Regressionsanalyse in Tab. 3 zielt auf das Studienpensum. Dafür wurden die Selbstauskünfte zum Studienpensum dichotomisiert. Das Modell richtet sich auf das absolvierte Pensum, das „viel weniger“ der Vorgabe der Studienordnung des derzeitigen Studiengangs entspricht. In die multivariate Modellrechnung wurden aus der Literatur bekannte studienbezogene Einflussfaktoren und der GALI eingeschlossen.

**Tab. 3:** Logistisches Regressionsmodell zum Studienpensum als abhängige Variablen (dichotomisiert)

Merkmale	Ausprägung	Modell Studienpensum^d^
		OR (95 %-KI)
Geschlecht	Männer	Ref
	Frauen	0,77 (0,599–0,989)^a^
Alter	–	1,04 (1,016–1,058)^c^
Deutsche Staatsangehörigkeit	Nein	Ref
	Ja	2,28 (1,186–4,393)^a^
Geburt in Deutschland	Nein	Ref
	Ja	1,71 (1,026–2,856)^a^
1. Fachsemester	Nein	Ref
	Ja	0,46 (0,328–0,643)^c^
Angestrebter Abschluss	Lehrerbildende Studiengänge, Master, Künstlerischer Abschluss, Diplom, Sonstige	Ref
	Bachelor	1,74 (1,351–2,250)^c^
Studienfächergruppe	Rechts‑, Wirtschafts- und Sozialwissenschaften	Ref
	Mathematik und Naturwissenschaften	1,83 (1,248–2,674)^b^
	Agrar‑, Forst- und Ernährungswissenschaften	1,23 (0,729–2,065)
	Ingenieurwissenschaften	1,13 (0,821–1,564)
	Sonstige	0,80 (0,567–1,125)
Abschlusszeugnis mit Studienzugangsberechtigung	Durchschnittsnote	1,40 (1,148–1,710)^b^
Finanzielle Belastung der Studierenden und Familie bis Studiumsabschluss	Gar nicht/kaum/etwas	Ref
	Ziemlich/sehr	1,68 (1,317–2,137)^c^
Erwerbstätig (neben Studium)	Nein	Ref
	Ja	1,16 (0,887–1,515)
„Global activity limitation indicator“ (GALI)	Keine gesundheitsbedingten alltäglichen Einschränkungen	Ref
	Mäßig eingeschränkt	1,56 (1,068–2,272)^a^
	Stark eingeschränkt	2,81 (1,643–4,802)^c^
Pseudo‑R^2^ (Nagelkerkes)	–	0,113
Cox & Snell R‑Quadrat	–	0,065
−2 Log-Likelihood	–	1.817,76
Hosmer-Lemeshow-Test	–	χ^2^ = 10,593 (df = 8)
*n*	–	2290

*OR *Odds Ratio, *KI *Konfidenzintervall

^a^*p* < 0,05

^b^*p* < 0,01

^c^*p* < 0,001

^d^Absolviertes Studienpensum dichotomisiert auf „viel weniger“ als die Vorgaben der Studienordnung im derzeitigen Studiengang (vs. etwas weniger/etwa genau so viel/etwas mehr/viel mehr)

Die multivariate Betrachtung ergibt, dass die Wahrscheinlichkeit eines viel geringeren Studienpensums für Frauen im Verhältnis zu Männern signifikant um den Faktor 0,77 (95 %-KI 0,60–0,99) erniedrigt ist. Daneben zeigt sich ein marginaler Einfluss des Alters auf das Studienpensum. Für die Studierenden gehen sowohl eine deutsche Staatsbürgerschaft als auch der Geburtsort in Deutschland mit einer signifikant erhöhten OR von 2,28 (95 %-KI 1,19–4,39) bzw. 1,71 (95 %-KI 1,03–2,86) einher. Die Wahrscheinlichkeit, dass das erreichte Studienpensum zum Befragungszeitpunkt viel weniger unter der Vorgabe der Studienordnung liegt, ist im 1. Fachsemester mit einer OR von 0,46 (95 %-KI 0,33–0,64) nur ca. halb so groß wie in den höheren Fachsemestern. In den Studiengängen mit Bachelorabschluss ist sie im Vergleich zu den übrigen angestrebten Abschlüssen signifikant erhöht (OR = 1,74; 95 %-KI 1,35–2,25). Die Studienfächergruppe Mathematik und Naturwissenschaften ist ebenfalls mit einem zu geringen Studienpensum assoziiert (OR = 1,83; 95 %-KI 1,25–2,67). Schlechtere Durchschnittsnoten im Abschlusszeugnis der Studienzugangsberechtigung korrespondieren mit einer größeren Vorhersagekraft, dass das erreichte Studienpensum zum Befragungszeitpunkt viel weniger als der Vorgabe entspricht. Einen bedeutsamen Einfluss nimmt in diesem Modell auch die Finanzlage ein, wenn die Studierenden und ihre Familien finanziell bis zum Studiumsabschluss ziemlich oder sehr belastet sind (OR = 1,68; 95 %-KI 1,32–2,14).

Ein Hauptergebnis dieser multivariaten Analyse ist, dass der GALI als stärkste Einflussvariable identifiziert wird. Je schwerer sich die gesundheitlich bedingte Einschränkung von Studierenden auf ihre alltäglichen Aktivitäten auswirkte, desto geringer war das bislang absolvierte Pensum im derzeitigen Studiengang. Die Wahrscheinlichkeit eines viel geringeren Studienpensums war für Studierende, die gesundheitsbedingt bei alltäglichen Aktivitäten mäßig eingeschränkt waren, um 1,56-fach (95 %-KI 1,07–2,27) und für Studierende mit gesundheitsbedingt starken Einschränkungen um 2,81-fach (95 %-KI 1,64–4,80) gesteigert.

Bei diesem logistischen Modell sind die überprüften Korrelationen zwischen den Prädiktoren gering ausgefallen, was darauf hindeutet, dass Multikollinearität die Regressionsanalysen nicht konfundiert hat. Linearität wurde mit dem Box-Tidwell-Verfahren überprüft und kann für alle Variablen angenommen werden. Die „goodness of fit“ wurde mit dem Hosmer-Lemeshow-Test überprüft, der eine hohe Anpassungsgüte anzeigt. Es ist jedoch eine geringe Modellgüte auf Basis des Bestimmtheitsmaßes zu konstatieren. Das binäre logistische Regressionsmodell war statistisch signifikant, aber die Varianzaufklärung von 11,3 % (Nagelkerkes R^2^) war gering.

## Diskussion und Fazit

Es ist vorab auf Limitationen von „computer assisted web interviews“ hinzuweisen. Diese Befragungsmethode erfasst nur Selbstauskünfte und die subjektiv wahrgenommene Gesundheit ohne parallele medizinische Untersuchungen. Die Erhebung war für die Studierenden freiwillig und die Response war unter Pandemiebedingungen nicht hoch. Außerdem könnten unterschiedliche Rücklaufquoten zwischen Gruppen und Fachbereichen die Ergebnisse zu einem gewissen Grad konfundiert haben. Der höheren Beteiligung von Frauen wurde methodisch durch Stratifizierung der Gesundheitsergebnisse nach Geschlecht und Adjustierung in der Modellierung begegnet. Es ist zu berücksichtigen, dass es sich um eine Querschnittserhebung handelt, die methodisch keine Ursache-Wirkungs-Beziehung bestimmen kann. Dennoch können enge Assoziationen als Indizien dafür angesehen werden, die wichtige Hinweise für weitere Forschung geben. Eine Wiederholungsbefragung ist an der Universität Kassel in Vorbereitung.

Ein Fünftel der Studierenden schätzte den allgemeinen Gesundheitszustand als mittelmäßig, schlecht oder sehr schlecht ein. Dieses Ergebnis liegt über allen eingangs erwähnten Gesundheitssurveys [8, 11], die zu früheren Zeitpunkten durchgeführt wurden. Eine Verschlechterung des subjektiven Gesundheitszustands ist aufgrund der Coronapandemie anzunehmen und stünde im Einklang mit dem Forschungstand [31]. Dagegen liegen die Anteile der Studierenden mit chronischen Krankheiten sowohl bei Männern als auch bei Frauen etwas unter den Durchschnittswerten der repräsentativen GEDA 2019/2020-EHIS-Studie des RKI für die Altersgruppe von 18 bis 29 Jahren [11]. Dies trifft ebenso auf die gesundheitsbedingten Einschränkungen bei alltäglichen Aktivitäten zu [11].

Besonders auffällig sind die Prävalenzraten der psychischen Erkrankungen unter Studierenden und darunter insbesondere bei Frauen. Mit der gesellschaftlichen Entstigmatisierung von psychischen Erkrankungen ist die „Symptomaufmerksamkeit“ gestiegen, aber möglichweise ist sie bei Frauen stärker als bei Männern ausgeprägt. Diese Haupttrends wurden schon in früheren Untersuchungen bei Studierenden vor der Coronapandemie in Deutschland beobachtet [7, 8, 14, 20]. Das deutet auf einen Zusammenhang zwischen Gesundheit und relevanten studienbezogenen Faktoren hin [34]. Studierende mit einem hohen Stresserleben im Studium bekunden vermehrt psychosomatische Beschwerden sowie eine negative Gesundheit [30]. Insgesamt weisen die Vergleichsanalysen auf die Bedarfslagen vulnerabler Gruppen, mögliche Verschlechterungen in der Coronapandemie und das Potenzial für die Prävention und Gesundheitsförderung hin.

Außerdem ist bei allen Vergleichen mit bundesweiten oder hessischen Erhebungen davon auszugehen, dass Studierende der Universität Kassel aufgrund des regionalen Einzugsgebiets per se stärker gesundheitlich belastet sind. Der hessische Landessozialbericht [12] weist für das ökonomisch deprivierte Nordhessen eine vergleichsweise ungünstige Gesundheitslage der Wohnbevölkerung aus.

Die vorliegende Querschnittsanalyse zeigt eine enge Assoziation zwischen dem GALI und dem selbstberichteten Studienpensum auf. Sie ist nach unserem Kenntnisstand die erste empirische Studie, die diesen Zusammenhang untersucht. Gesundheit und Arbeitsfähigkeit sind Voraussetzungen, um Arbeitsaufgaben zu einem bestimmten Zeitpunkt zu bewältigen [10, 18]. Die Auswertungen belegen große Schwierigkeiten im Umgang mit relevanten Studienanforderungen aus der Perspektive der Studierenden. Bei allen abgefragten Anforderungen nahm der Anteil der Studierenden mit diesen Schwierigkeiten mit der Stärke der Gesundheitseinschränkungen zu. In multivariater Betrachtung war bei Studierenden mit starken gesundheitsbedingten Einschränkungen bei alltäglichen Aktivitäten die Wahrscheinlichkeit, dass das absolvierte Studienpensum viel weniger als die Vorgabe der Studienordnung im derzeitigen Studiengang ausmacht, um den Faktor 2,81 gesteigert. Das OR ist damit fast doppelt so hoch wie bei den gesundheitsbedingt mäßig eingeschränkten Studierenden.

Die festgestellten Assoziationen zwischen Studium und Gesundheit sind jedoch differenziert zu betrachten. Trotz der signifikanten Gesundheitsunterschiede berichteten mehr Männer als Frauen, dass ihr absolviertes Studienpensum viel weniger den Vorgaben der Studienordnung im derzeitigen Studiengang entsprechen würde. Bei den im Durchschnitt gesünderen ausländischen Studierenden ist daneben ein „healthy migrant effect“ [27] vorstellbar. Selektionseffekte könnten entstehen, wenn sich ausländische Studierende mit Krankheiten beispielsweise ein Studium in Deutschland oder die Reisemöglichkeiten unter den Bedingungen der Coronapandemie nicht zutrauen würden.

Als interessanter Befund kristallisierte sich in den multivariaten Analysen heraus, dass Studierende in den Studiengängen mit Bachelorabschluss größere Probleme haben, das vorgegebene Studienpensum zu erreichen, als in den anderen Studiengängen. Eine Ausnahme stellt jedoch das 1. Fachsemester dar. Dies deutet darauf hin, dass es sich in der Prävention lohnen könnte, in der Eingangsphase in den Studiengängen mit Bachelorabschluss (noch) stärker anzusetzen und die bestehenden Unterstützungs- und Informationsangebote von Studierendenwerk und Hochschule besser zu vernetzen. Wie an anderer Stelle von Hollederer et al. [16] untersucht, sind Bekanntheitsgrad und Nutzung der vorhandenen Beratungs- und Unterstützungsangebote bei Studierenden mit chronischen Erkrankungen eher gering. Generell ist an den Hochschulen ein strukturiertes studentisches Gesundheitsmanagement (SGM) flächendeckend zu entwickeln [1, 22]. Es sollte sowohl Gesundheitsförderungsmaßnahmen mit großer Reichweite auf dem Campus, aber auch idealerweise ein Gesundheitsmanagement für Studierende mit amtlich anerkannten Behinderungen und starken gesundheitsbedingten Einschränkungen beinhalten. Ein Fallmanagement könnte möglicherweise schon bei Einwahl in die Studiengänge oder im Studienverlauf einsetzen, wenn Nachteilsausgleiche von Studierenden in Anspruch genommen werden [15]. Der gleichberechtigte Zugang zu allgemeiner Hochschulbildung ist nicht nur ein Recht von Menschen mit Behinderungen nach der UN-Behindertenrechtskonvention [2], es profitieren auch die Hochschulen selbst, wenn sie bei aktuell zurückgehenden Studierendenzahlen wieder stärker nachgefragt und die laufenden Studiengänge erfolgreicher abgeschlossen werden.

## Fazit für die Praxis

- In Deutschland nimmt die Zahl der Menschen mit Behinderungen insgesamt, aber auch unter Studierenden und damit an Hochschulen zu.
- Bisherige Gesundheitssurveys zeigen, die Gesundheit von Studierenden ist nicht so gut wie erwartet.
- Besonders auffällig sind die Prävalenzraten von psychischen Erkrankungen unter Studierenden, insbesondere bei Frauen.
- Der Gesundheitszustand von Studierenden ist eng mit Schwierigkeiten bei den Anforderungen im Studium und dem Studienpensum assoziiert.
- Die Ergebnisse haben Implikationen für Nachteilsausgleiche, Informations- und Beratungsangebote, Gesundheitsförderung und Barrierefreiheit an Hochschulen.
- Es braucht ein strukturiertes studentisches Gesundheitsmanagement (SGM).
- Der Aufbau einer systematischen Gesundheitsberichterstattung für Studierende wird empfohlen.
